# FOXO3 directly regulates an autophagy network to functionally regulate proteostasis in adult neural stem cells

**DOI:** 10.1371/journal.pgen.1008097

**Published:** 2019-04-11

**Authors:** Amanda J. Audesse, Shleshma Dhakal, Lexi-Amber Hassell, Zachary Gardell, Yuliya Nemtsova, Ashley E. Webb

**Affiliations:** 1 Neuroscience Graduate Program, Brown University, Providence, Rhode Island, United States of America; 2 Department of Molecular Biology, Cell Biology and Biochemistry, Brown University, Providence, Rhode Island, United States of America; 3 Center on the Biology of Aging, Brown University, Providence, Rhode Island, United States of America; 4 Carney Institute for Brain Science, Brown University, Providence, Rhode Island, United States of America; Weill Cornell Medical College, UNITED STATES

## Abstract

Maintenance of a healthy proteome is essential for cellular homeostasis and loss of proteostasis is associated with tissue dysfunction and neurodegenerative disease. The mechanisms that support proteostasis in healthy cells and how they become defective during aging or in disease states are not fully understood. Here, we investigate the transcriptional programs that are essential for neural stem and progenitor cell (NSPC) function and uncover a program of autophagy genes under the control of the transcription factor FOXO3. Using genomic approaches, we observe that FOXO3 directly binds a network of target genes in adult NSPCs that are involved in autophagy, and find that FOXO3 functionally regulates induction of autophagy in these cells. Interestingly, in the absence of FOXO activity, aggregates accumulate in NSPCs, and this effect is reversed by TOR (target of rapamycin) inhibition. Surprisingly, enhancing FOXO3 causes nucleation of protein aggregates, but does not increase their degradation. The work presented here identifies a genomic network under the direct control of a key transcriptional regulator of aging that is critical for maintaining a healthy mammalian stem cell pool to support lifelong neurogenesis.

## Author summary

The buildup of protein aggregates is deleterious to cellular function and can cause neurodegenerative disease. Healthy cells use a process known as autophagy to degrade aggregates and remove damaged proteins and organelles as needed. This process is particularly important in stem cells, which must clear damaged cellular material to prevent its inheritance down the lineage. The mechanisms that control overall levels of autophagy in stem cells are not well understood. Here, we show that a transcriptional regulator, FOXO3, which is critical for supporting stem cell functionality, regulates a genomic network of autophagy genes in mouse neural stem and progenitor cells. We find that FOXO3 functions as a switch to induce autophagy in stem cells, and that its activity is required to restrain aggregate accumulation in these cells. This work is the first to elucidate a genomic program in neural stem cells that promotes aggregate clearance. Understanding how stem cells maintain protein quality control has important implications for using regenerative medicine to understand and treat age-related and degenerative diseases.

## Introduction

Cellular proteostasis, or maintenance of a healthy proteome, is critical for cellular homeostasis throughout life. Loss of proteostasis has been linked to aging, neurodegeneration and reduced regenerative capacity [1]. Age-related diseases of the brain in particular (e.g. Alzheimer’s disease, Fronto-temporal dementia, Parkinson’s disease) are associated with aggregate accumulation, and disruptions in basal proteostasis can cause neurodegeneration in mouse models [2, 3]. Normal, non-pathological aging is also associated with the buildup of protein aggregates across tissues, and clearance of these aggregates through the activation of autophagy can extend lifespan in some species [4].

Macroautophagy, hereafter referred to as autophagy, is a conserved catabolic pathway that functions to deliver cellular components to the lysosome for degradation by acid hydrolases [5]. Autophagic activity is present at low levels basally, and can be induced by cellular stress, nutrient deprivation, inhibition of TOR (target of rapamycin) signaling, or other cues. The process of autophagy begins with the formation of a phagophore, which acts as scaffold for protein and organelle recruitment. The phagophore elongates and eventually completely engulfs the cargo, forming an enclosed autophagosome. Fusion of the autophagosome with the acidic lysosome to form an autolysosome results in cargo degradation. This process requires a number of proteins comprising the autophagic machinery, which coordinately regulate each step of the pathway according to the needs of the cell. Dysregulation of autophagy in either direction is deleterious to basal cellular function and impairs the cell’s ability to adjust to changing environmental conditions.

Neural stem cells (NSCs) in the developing and adult mammalian brain have been shown to rely on active autophagic flux for functional support, including self-renewal and neurogenic capacity. In early development, activity of ATG5, a protein required for early autophagosome formation, functions to suppress proliferation and promote cortical neurogenesis [6]. Impairment of the autophagy pathway through genetic ablation of *Rb1cc1*/FIP200, a protein required for autophagosome formation, in the postnatal mouse brain causes loss of the NSC reservoir and reduced neurogenesis [7]. Recent work showed that autophagy and lysosome-associated activity is essential for NSCs in adult mice to re-enter the cell cycle [8]. Moreover, during aging, defects in the lysosome-autophagy pathway in the quiescent NSC pool in vivo leads to aggregate accumulation in these cells and impairs their ability to activate and proliferate. Importantly, these defects are reversible in aged animals through stimulation of the lysosome-autophagy pathway [8]. Thus, understanding the mechanisms regulating autophagy in the developing, adult, and aged brain is important for advancing our understanding of a broad range of neurological conditions, ranging from neurodevelopmental disorders to age-related neurodegeneration.

The FOXO family of transcription factors are critical regulators of cellular homeostasis, aging and stem cells [9, 10]. The FOXO family includes four members with overlapping functions: FOXO1, FOXO3, FOXO4 and FOXO6 [11]. Previous work demonstrated that the FOXOs, and FOXO3 in particular, function to maintain NSC homeostasis in mice. Ablation of *Foxo3* alone, or *Foxo1*, *Foxo3* and *Foxo4* in combination causes a depletion of the NSC pool in adult mice [12, 13]. In other systems, FOXOs have been observed to regulate protein quality control, including autophagy and the ubiquitin proteasome system [14]. The extent to which FOXO3 regulates proteostasis as a means to support NSC function remains unclear. Here, we identify FOXO3 as a key regulator of autophagy in NSCs. We find that FOXO3 promotes autophagy in NSPCs, and find it directly regulates this process through a network of autophagy genes. Moreover, we identify aggregated proteins as a cargo regulated by FOXO3-induced autophagy in NSPCs.

## Results

### FOXO3 directly binds and regulates a network of autophagy genes in adult neural stem and progenitor cells

**Fig 1:**
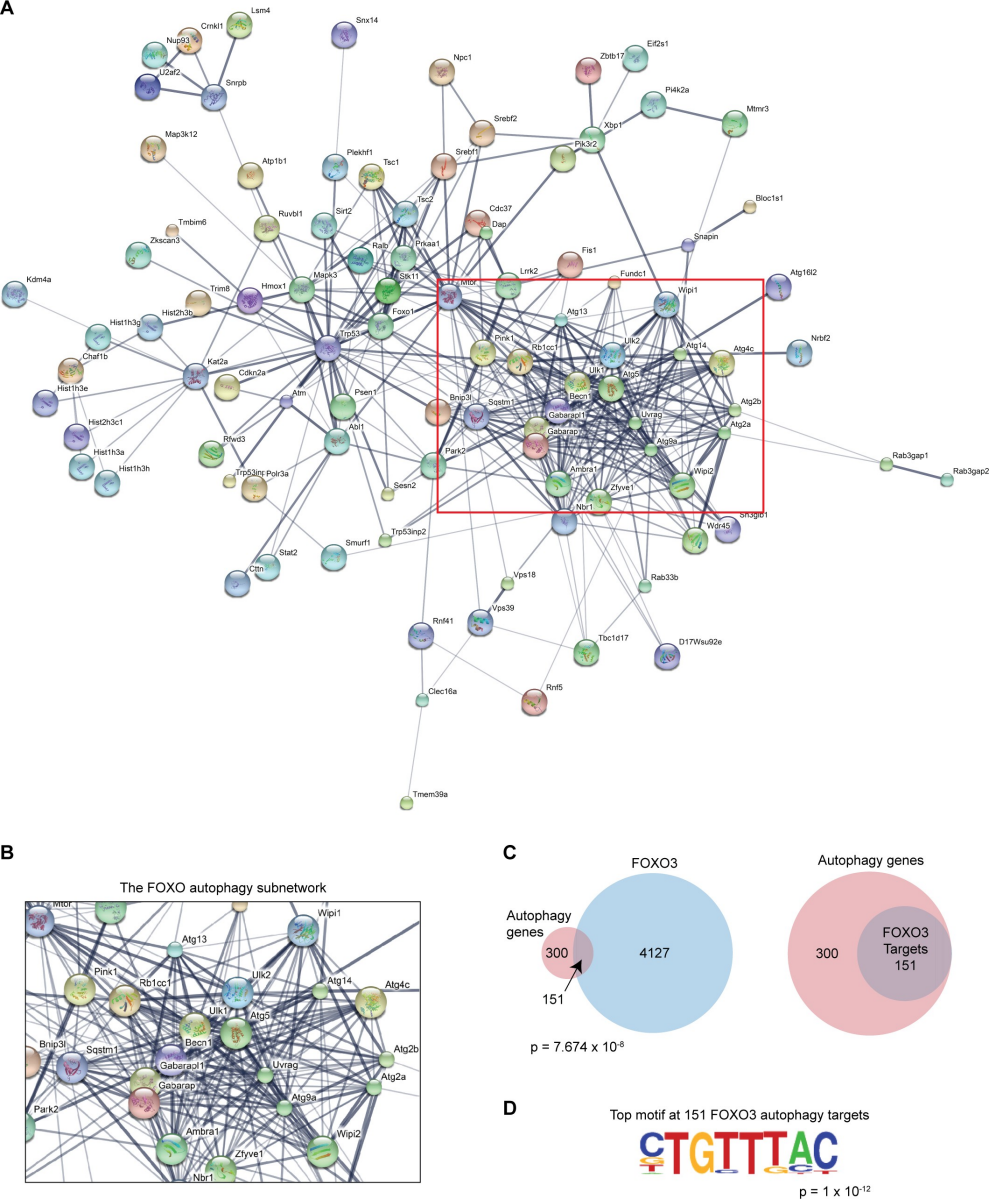
FOXO3 directly binds a network of autophagy genes in NSPCs. (A) STRING analysis of the direct FOXO3 autophagy-related network in NSPCs based on ChIP-seq analysis of FOXO3 binding in this cell type. (B) A snapshot of the highly connected autophagic machinery network (red box in 1A). (C) Overlap between FOXO3 bound genes based on ChIP-seq analysis and autophagy genes. Left panel shows enrichment of autophagy genes among all FOXO3 targets and right panel depicts the FOXO3 targets within the entire autophagy network. p-value from Fisher’s exact test is shown. (D) Motif analysis of FOXO3 binding sites at the 151 direct autophagy targets. The top most significant motif enriched in the analysis is shown, which corresponds to the FOXO consensus binding motif.

We hypothesized that FOXO3 could directly regulate the autophagy pathway at the transcriptional level. Thus, we sought to identify the complete program of autophagy genes downstream of FOXO3 in primary adult neural stem and progenitor cells (NSPCs; note we refer to cultured primary NSCs as NSPCs because they contain a mix of stem and progenitor cells). To do so, we analyzed our previously generated FOXO3 ChIP-seq data from adult (12 week) NSPCs, in which we identified 4278 direct FOXO3 targets [15, 16]. Specifically, we investigated the enrichment of an autophagy gene set (GO:0006914) containing 451 unique genes, including factors involved in the regulation of autophagy, cargo recruitment, vesicle trafficking and elongation, and autophagosome assembly. To examine the connectivity among the direct FOXO3 targets in the autophagy pathway, we performed a network analysis using STRING [17] (Fig 1A). We observed that among the broad FOXO3 autophagy network, there was an extensively connected subnetwork that included the autophagic machinery (Fig 1A and 1B). Direct overlap between the FOXO3 ChIP-seq targets and the autophagy network revealed a significant enrichment of FOXO3 targets among autophagy related genes: 151/451 autophagy genes were direct FOXO3 targets in NSPCs (p = 7.674 x 10^−8^ by Fisher’s exact test; Fig 1C and S1 Table). Notably, motif analysis of the 151 direct FOXO3 autophagy genes revealed that the most highly enriched sequence motif at these sites was the FOXO3 consensus motif (TGTTTAC; p = 1 x 10^−12^; Fig 1D). This finding suggests that FOXO3 directly functions as a central node in the regulation of autophagy in NSPCs.

**Fig 2:**
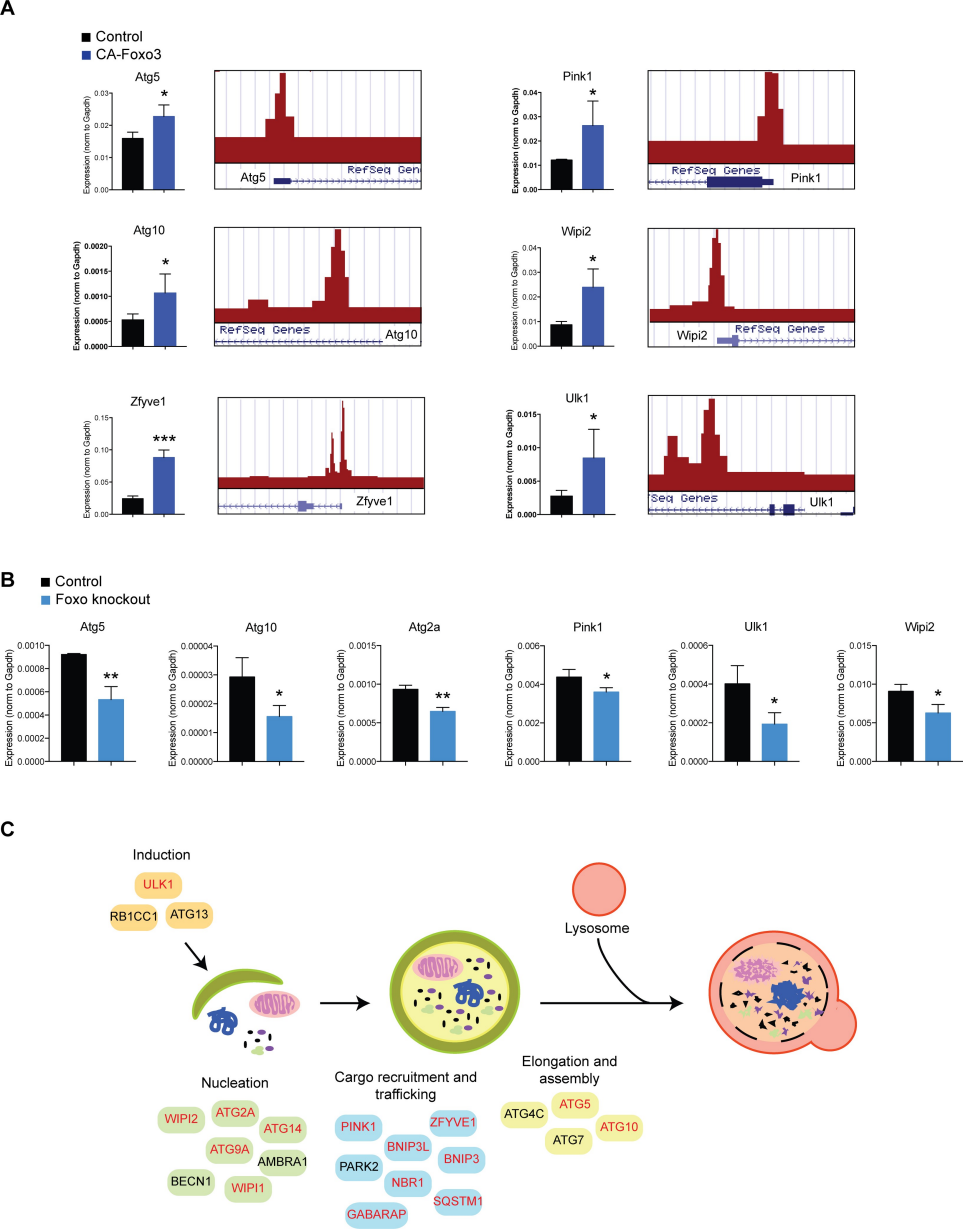
FOXO transcription factors regulate autophagy genes in NSPCs. (A) Examples of FOXO3 target genes that are induced by FOXO3. For each target, RT-qPCR analysis showing induction by CA-FOXO3 is on the left, and a snapshot of the genome browser showing FOXO3 binding enrichment (ChIP-seq in NSPCs) is shown on the right. (B) Ablation of FOXO transcription factor in NSPCs reduces expression of autophagy genes. For (A) and (B), n = 3 experiments; Student’s t-test; *p < 0.05, **p < 0.01, ***p < 0.001. (C) Schematic diagram of the autophagy pathway showing direct FOXO3 targets identified in this study and their role in autophagy. Genes shown in red were also induced by FOXO3 in this study.

We next asked if FOXO3 was sufficient to regulate the target genes observed in the FOXO3 network. We first took an overexpression approach to test the hypothesis that FOXO3 could induce expression of genes in the autophagic machinery subnetwork. We used a doxycycline-inducible lentivirus system to overexpress FOXO3 in primary NSPCs for 24 hours, followed by reverse transcription and quantitative PCR (RT-qPCR) analysis of candidate target genes. We used a constitutively active form of FOXO3 (CA-FOXO3) that lacks the three AKT phosphorylation sites because it localizes to the nucleus in cultured NSPCs, which recapitulates the localization of FOXO3 in vivo [12, 13]. We tested 23 candidate genes in the autophagic subnetwork and observed that 16 genes were significantly induced by CA-FOXO3 (p < 0.05, Student’s t-test; Fig 2A and S1 Fig). The targets that were induced by FOXO3 are involved in the autophagy initiation system (e.g. *Rbcc1* and *Ulk1*), autophagosome formation and elongation (e.g. *Atg14*, *Gabarap*, *Atg5*, *Atg10*), autophagosome recycling (e.g. *Wipi1*, *Wipi2*, *Atg9a*) and cargo recruitment (*Sqstm1*). Notably, some direct FOXO3-bound targets in the ChIP-seq analysis were not induced by FOXO3 (e.g. *Ambra1*, *Atg2b* and *Atg4c*), suggesting that they require additional co-factors for induction under basal conditions. Nevertheless, FOXO3 has the capacity to induce a coordinated gene program regulating multiple processes in the autophagy pathway in NSPCs.

To complement this overexpression approach, we also investigated the extent to which these genes were downregulated upon ablation of FOXOs. Previous work showed that three closely related FOXO transcription factors (FOXO1, FOXO3 and FOXO4) function partially redundantly in adult mouse NSCs and other cell types [12, 18]. Therefore, we ablated all three FOXO transcription factors in NSPCs and examined the effect on expression of the putative FOXO target genes. We isolated NSPCs from mice harboring floxed alleles for *Foxo1*, *Foxo3*, and *Foxo4* (we refer to this mouse line as “Trifloxed”). To ablate the *Foxo* genes, we infected Trifloxed NSPCs with an adenovirus carrying Cre-Recombinase, or a control adenovirus (empty vector or GFP). We confirmed targeting of the three loci using western blot and RT-qPCR analysis (S2 Fig). We then performed RT-qPCR analysis of the candidate autophagy genes in the FOXO Trifloxed NSPCs. We observed that six targets were significantly downregulated in the FOXO-ablated cells, including *Atg5*, *Atg10*, and *Pink1* (Fig 2B). To gain a more global view of how autophagy networks are altered in the absence of FOXO3, we performed an analysis of previously published microarray expression data from wild type and FOXO3 null adult NSPCs [13]. We observed either up or downregulation of a number of autophagy genes in this context (S2C Fig and S3 Table), including direct FOXO3 targets. Thus, cells that lack *Foxo3* since birth also exhibit expression changes in the autophagy gene network, and this dysregulation includes both direct and indirect targets of FOXO3, likely due to indirect feedback within the gene network. Together, our results indicate that FOXOs directly regulate autophagy genes at the transcriptional level in NSPCs.

In addition to promoting molecular quality control, the autophagy pathway functions to turnover damaged mitochondria. This selective form of autophagy is known as mitophagy. One of the strongest FOXO3 targets in our expression data was *Pink1*, a kinase that functions as a molecular sensor of damaged mitochondria (Figs 1B, 2A and 2B) [19]. We asked if FOXO3 could more generally regulate mitophagy genes in NSPCs and observed significant overlap with the mitophagy gene ontology gene set (GO:0000422). Out of 83 total genes in the mitophagy pathway, 43 genes (51.8%) were bound by FOXO3 in ChIP-seq analysis in NSCs (p = 6.77 x 10^−9^, Fisher’s exact test, S3A Fig). We examined expression of a subset of these targets in FOXO-ablated and FOXO3-overexpressing cells and identified several new targets (e.g. *Fis1* and *Optn*) that were modulated compared to basal conditions (S3B and S3C Fig). To further test whether mitophagy might be defective in FOXO-ablated cells, we examined PINK1 protein levels by western blot. We found that PINK1 protein was reduced in the absence of FOXOs, and induction of autophagy by starvation stress (HBSS) was not sufficient to rescue PINK1 levels (S3D Fig). Thus, in addition to regulating autophagy, FOXO3 regulates genes involved in mitophagy and may be a critical regulator of mitochondrial turnover in NSPCs.

### FOXO3 induces autophagic flux in NSPCs

**Fig 3:**
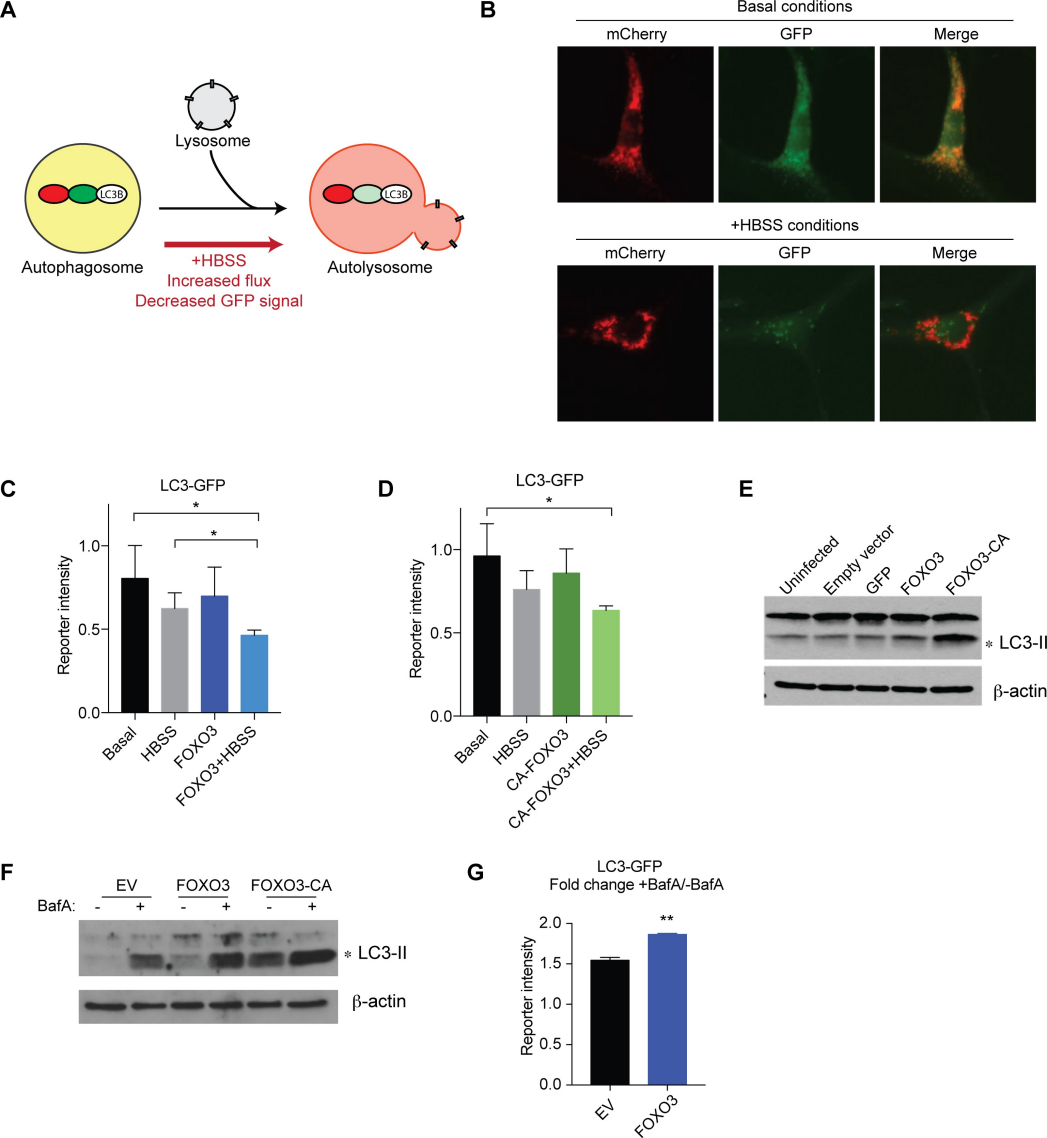
FOXO3 regulates autophagy in NSPCs. (A) Schematic representation of the tandem LC3 reporter system. The processed LC3 reporter localizes to autophagosomes and fluoresces both red and green. Upon fusion with the acidic lysosome, the low pH of the autolysosome quenches the GFP fluorescence, but the mCherry fluorescence remains stable. Under starvation conditions (+HBSS), flux through the autophagic pathway increases, resulting in reduced GFP signal. (B) Example images of the tandem LC3 reporter in NSPCs under basal conditions (upper panels) and starvation conditions (two hours in HBSS; lower panels). Note the reduced GFP signal in the +HBSS condition. (C) Overexpression of FOXO3 further enhances autophagic flux under HBSS conditions, as indicated by a decrease in GFP signal. (D) Similar to wild type FOXO3, constitutively active FOXO3 enhances autophagic flux under starvation conditions. n = 3 experiments; *p < 0.05. (E) Overexpression of wild type or constitutive active FOXO3 increases endogenous LC3 levels as shown by western blot in NSPCs. (F) BafA treatment further enhances processed LC3 levels upon FOXO3 or CA-FOXO3 overexpression. One representative example of at least three replicates is shown for (E) and (F). (G) Overexpression of wild type FOXO3 increases autophagy induction. Fold change of LC3-GFP buildup in the presence of BafA compared to basal conditions is shown. n = 3 experiments; **p < 0.01.

The observation that FOXO3 regulates a network of autophagy genes in NSPCs led us to investigate the extent to which FOXO3 can induce autophagic flux in these cells. To do so, we used an mCherry-GFP-LC3 tandem reporter (Fig 3A and 3B) [20]. LC3 (microtubule-associated protein light chain 3) is a member of the *Atg8* gene family, which encodes proteins involved in autophagosome assembly, maturation, and fusion with lysosomes. Processed LC3 protein (LC3-II) stably associates with autophagosome membranes and is a commonly used marker of the autophagy pathway. This reporter takes advantage of the differential sensitivity of GFP and mCherry to low pH. GFP is sensitive to low pH and loses fluorescence in acidic conditions, such as in the lysosomal compartment. In contrast, mCherry retains fluorescence at low pH. Upon induction of autophagy, for example in response to two hours starvation in HBSS, GFP signal is reduced due to increased flux (Fig 3B, S4 and S5A Figs), and blocking autophagy strongly induces GFP and mCherry puncta (S4 Fig). Thus, the reporter system can be used to measure changes in autophagic flux based on changes in GFP levels compared to the overall level of autophagy (autophagosomes and autolysosomes marked by red). We infected primary adult NSPCs with lentiviral FUW-mCherry-GFP-LC3 and used fluorescence activated cell sorting (FACS) to determine levels of autophagic flux under basal conditions, in response to starvation (two hours HBSS treatment), upon overexpression of wild type FOXO3, or in response to starvation and FOXO3 overexpression in combination. We used doxycycline-inducible lentiviruses to overexpress FOXO3 for 24 hours. We found that overexpression of wild type FOXO3 modestly (though not statistically significantly) decreased GFP intensity in this assay (Fig 3C and S5A and S5B Fig). In contrast, starvation combined with FOXO3 overexpression significantly lowered GFP reporter levels compared to basal conditions or starvation alone (Fig 3C and S5C Fig), indicating enhanced autophagic flux under these conditions. We observed a similar response with a constitutively active form of FOXO3 (CA-FOXO3) in combination with starvation (Fig 3D and S5D Fig). We did not observe changes in the intensity of mCherry under these conditions (S5E and S5F Fig), suggesting that the overall level of the reporter and overall autophagosome formation did not change under the different conditions. Together these observations indicate that elevated FOXO3 levels can enhance flux through the autophagy pathway under starvation conditions.

To determine if FOXO3 can increase levels of endogenous autophagy, we used doxycycline-inducible lentiviruses to overexpress FOXO3 in NSPCs as described above and examined levels of the autophagy protein LC3 (microtubule-associated protein light chain 3) by western blot. We found that 24 hours after induction with doxycycline, endogenous LC3 levels were elevated in cells overexpressing wild type FOXO3, and more strongly induced by CA-FOXO3 (Fig 3E). To confirm that elevated LC3 was due to induction of autophagy and not a decrease in degradation, we performed the same experiment overexpressing FOXO3, CA-FOXO3, or EV control virus in NSPCs, and treated the cells with BafilomycinA to block fusion of the autophagosome to the lysosome, thereby blocking autophagy after induction. We observed that processed LC3 was further enhanced in the BafA treated cells compared to basal conditions, particularly in the FOXO3 and FOXO3-CA overexpressing cells (Fig 3F). Finally, we performed an overexpression experiment using the reporter system including blocking the pathway using BafA and observed a similar accumulation of the reporter (Fig 3G). Thus, FOXO3 overexpression increases levels of the autophagosome-associated form of LC3, (LC3-II) suggesting that FOXO3 can enhance endogenous levels of processed LC3 in primary NSPCs and increases autophagy induction in these cells.

### Ablation of FOXOs in NSPCs disrupts autophagic turnover

**Fig 4:**
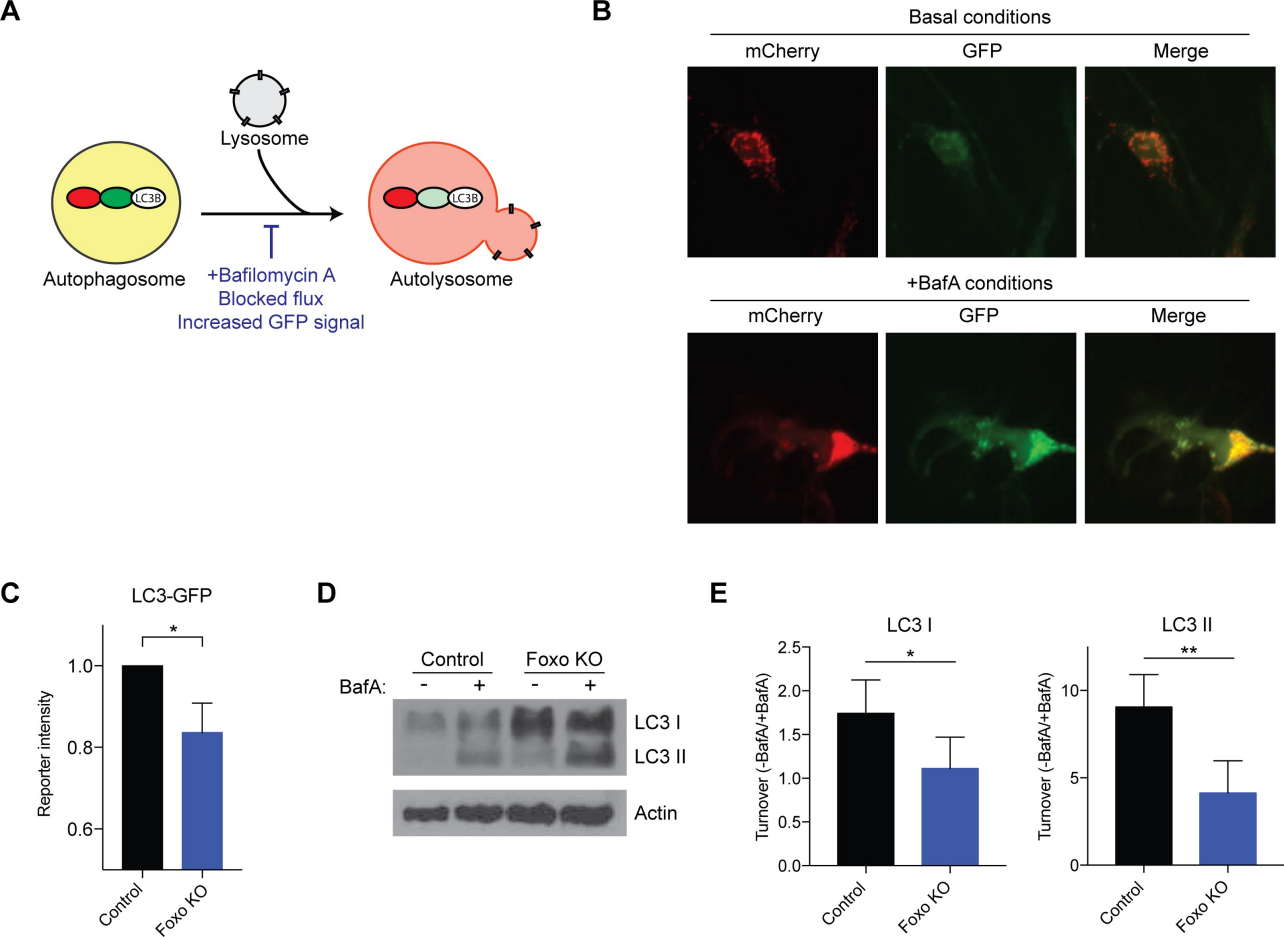
FOXOs are required to maintain autophagy levels in NSPCs. (A) Schematic representation of the LC3 tandem reporter system. Bafilomycin A disrupts proton transport in lysosomes and blocks fusion of the lysosome with the autophagosome. (B) Representative images of the LC3 reporter system under basal conditions and in response to Bafilomycin A. Note the increase in mCherry and GFP in the +Bafilomycin A conditions. (C) Ablation of FOXO transcription factors in NSPCs reduces autophagic flux. Quantification of FACS experiments detecting GFP intensity in control cells (empty vector Trifloxed cells) and FOXO-ablated cells (FOXO conditional KO cells) in the presence of Bafilomycin A. (D) FOXOs regulate the turnover of endogenous LC3 in NSPCs. Western blot showing levels of LC3 I and LC3 II in wild type and FOXO ablated NSPCs, with and without Bafilomycin A treatment to detect LC3 turnover. (E) Quantification of western blots shown in (D). LC3 turnover was reduced in the FOXO-ablated cells, indicating that FOXOs are required to maintain the endogenous level of autophagic turnover in NSPCs. n = 3 experiments for C-E; Student’s t-test; *p < 0.05, p** < 0.01.

We next tested the hypothesis that FOXO3 is required for maintenance of autophagy in NSPCs. To do so, we co-infected the FOXO Trifloxed cells with adenovirus (empty control or Cre-recombinase) together with the lentiviral mCherry-GFP-LC3 tandem reporter. Five days post-infection, we measured autophagic flux by FACS under basal conditions and in cells treated with Bafilomycin A, which prevents lysosomal acidification, thereby blocking flux (Fig 4A and 4B). We observed reduced autophagic flux in the FOXO-ablated Trifloxed NSPCs compared to control infected Trifloxed cells (Fig 4C and S5G and S5H Fig). In contrast, FOXO-ablation did not alter the response to starvation stress (HBSS) (S5I Fig). These findings are consistent with a role for FOXO transcription factors in maintaining levels of the autophagy pathway in NSPCs, and the idea that under acute starvation stress, NSPCs can bypass the requirement for FOXOs. We next examined the extent to which FOXOs regulate the turnover of endogenous LC3 in NSPCs. We used Bafilomycin A to block autophagic flux and measured turnover of LC3 I and LC3 II in wild type and FOXO ablated NSPCs. In both cases, LC3 turnover was reduced in the FOXO-ablated cells (Fig 4D and 4E), indicating that FOXOs are required to maintain the endogenous level of autophagic turnover in NSPCs.

### FOXOs regulate aggregate levels in NSPCs

**Fig 5:**
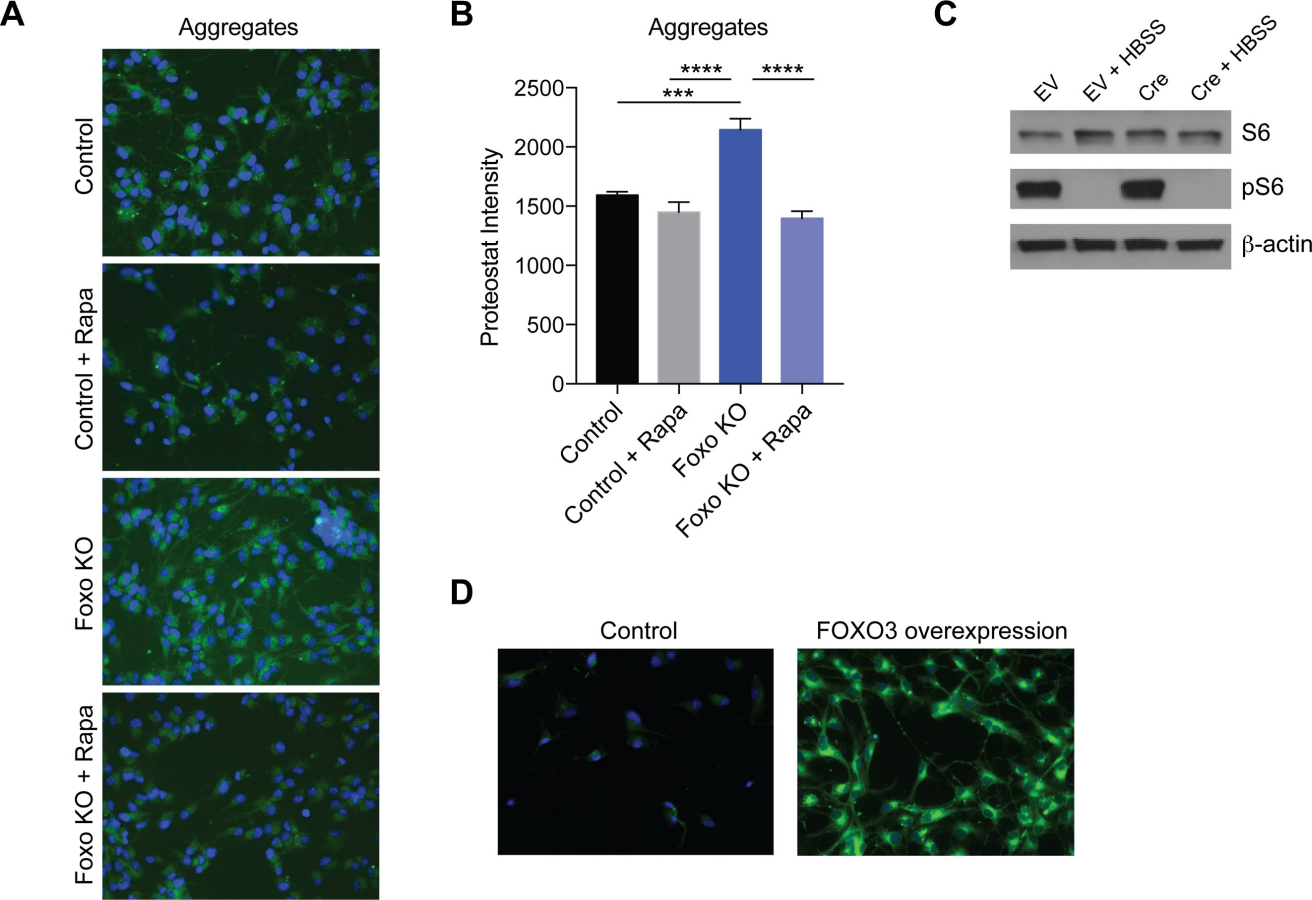
FOXO3 promotes aggregate clearance in NSPCs. (A) Protein aggregates are significantly increased in the absence of FOXO activity and this increase is reduced by TORC1 inhibition (24 hour rapamycin treatment). (B) Quantification of aggregate formation assay. TORC1 inhibition reverses the effect of FOXO ablation on aggregate levels. n = 3; Student’s t-test; ***p < 0.001, ****p < 0.0001. (C) Western blot showing no change in TOR activity in the absence of FOXOs in NSPCs, but reduced pS6 under starvation (HBSS) conditions. (D) Protein aggregates are increased in response to FOXO3 overexpression (example of n = 3 biological replicates).

To examine the functional consequences of altering autophagy through the inhibition of FOXO activity, we measured protein aggregate levels in control and FOXO-ablated NSPCs. We used proteostat, a fluorescent dye that specifically binds aggregated proteins to measure overall levels of aggregation. We found that NSPCs lacking FOXOs displayed significantly increased aggregate levels (Fig 5A and 5B). To investigate the mechanism by which FOXO ablation increased aggregate levels in NSPCs, we tested the hypothesis that increased TOR signaling may be responsible. The TOR pathway functions in nutrient and stress signaling, and reduction of TOR activity has been shown to induce autophagy [21]. Moreover, mTOR is a major hub in our STRING network analysis, with strong connectivity with autophagy genes (Fig 1A), and a recent study reported elevated TOR activity in FOXO-ablated neurons [22]. We found that the increased aggregation due to FOXO ablation was reduced back to wild type levels upon mTORC1 inhibition (24 hours rapamycin treatment; Fig 5B). This finding raises the possibility that FOXOs promote aggregate removal in NSPCs through a mechanism involving repression of mTORC1 activity. Alternatively, mTORC1 repression of autophagy could be independent of FOXO. To distinguish between these possibilities, we investigated mTORC1 activity levels in wild type and FOXO-ablated NSPCs under basal and starvation (HBSS) conditions. We found that phosphorylation of S6 was not changed upon ablation of FOXOs, either under basal or starvation conditions (Fig 5C). We conclude that in NSPCs under basal conditions, increased aggregate formation is not likely due to repression of autophagy due to increased TOR activity.

Finally, we asked whether intracellular aggregates could be cleared by FOXO3 overexpression. We compared levels of proteostat-positive aggregates in wild type NSPCs relative to FOXO3-overexpressing cells (24 hours acute overexpression). Surprisingly, we observed an acute increase in aggregates in the overexpression conditions (Fig 5D). This observation may reflect an increase in the loading of aggregates into the autophagy pathway, but a failure of the degradation machinery to process them. Thus, it is likely that additional factors are required for the complete turnover and clearance of aggregates.

## Discussion

Macroautophagy is essential for the maintenance of a healthy proteome in adult tissues. Disruptions in proteostasis, and the autophagy-lysosome system in particular, have been linked to aggregate accumulation and reduced neurogenesis in mammals [7, 8, 23]. In this work, we found that the FOXO3 transcription factor, which is a conserved determinant of the aging process [24–26], promotes autophagy in NSPCs through a direct network of autophagy genes. FOXO3 has been implicated in cellular quality control in other systems, including mature neurons [14, 22, 27], and more recently in neurogenesis [28]. Maintenance of cellular quality control is particularly important in stem cells, which may pass un-cleared aggregates to daughter cells and eventually terminally differentiated cells in the lineage. Consistent with our findings here, recent work shows that actively cycling NSPCs maintain high levels of autophagy and that this pathway is crucial for their activation [8]. Thus, an active autophagy pathway may be crucial to prevent transfer of damaged proteins or organelles to new neurons. Together, these findings suggest that FOXO3 is likely to be critical to coordinate a program of autophagy genes to support high rates of autophagy in adult NSCs and maintain the health of the neurogenic lineage.

Re-analysis of our previously published ChIP-seq data [15, 16] revealed that autophagy targets are highly enriched directly downstream of FOXO3. We also observed dysregulation of several of these targets in *Foxo3*-ablated animals. While there was a strong connection between direct FOXO3 binding and gene regulation at many of the targets observed here, we also note that a number of autophagy genes in the direct FOXO3 network were not transcriptionally regulated by FOXO3. The reason for this difference is unclear, but one interesting possibility is that additional co-factors are required for full activation of the FOXO3 autophagy network. For example, the bHLH transcription factor TFEB functions as a conserved transcriptional regulator of lysosomal-autophagy networks [29, 30]. FOXO3 has been shown to co-regulate gene expression with other bHLH factors such as ASCL1 and MYC [16, 31], as well as other families of transcription factors (e.g. β-catenin) [32]. Moreover, FOXO binding sites are highly enriched for motifs that could be engaged by other families of regulators [15]. Such synergistic control is likely to be critical for stringent regulation of autophagy levels to achieve optimal cellular quality control under changing environmental conditions. In future work, it will be particularly important to understand the interplay between FOXO3 and other transcriptional networks regulating autophagy, as manipulation of multiple factors may be necessary to fully activate the autophagy-lysosomal network for cargo turnover or clearance.

Our data indicate that the cellular function of FOXO3-mediated autophagy is to maintain cellular quality control, which is critical to maintain low levels of cellular aggregates. Clearance of aggregates is essential to support cellular homeostasis and prevent neurodegeneration during aging [33]. Whether there are specific types of aggregates that are targeted in NSPCs remains unknown. In future studies, it will be important to investigate the identity of the aggregates, as this is likely to shed light on the etiology of particular neurodegenerative diseases. For example, Aβ aggregates, which are the source of extracellular Aβ plaques in Alzheimer’s disease, can be degraded through the autophagy pathway [34]. Moreover, autophagy is impaired in Alzheimer’s disease patients [35]. The extent to which FOXO3 promotes Aβ clearance has not been explored but will be important to address in future studies. Recent work implicates FOXOs in α-synuclein turnover in the context of Parkinson’s disease. In this context, FOXO3 was found to protect dopaminergic neurons in the substantia nigra against α-synuclein oligomer accumulation [36, 37]. Whether FOXO-mediated protection in the context involves autophagy has not been investigated. Importantly, recent work has shown that FOXOs promote autophagy and are neuroprotective in neurons in the aging brain [22]. In this context, FOXOs appear to restrain TOR activity, either directly or indirectly, to maintain healthy levels of autophagy. It will be important to determine how FOXOs differentially regulate autophagy in different cell types in the brain to restrain aggregate accumulation.

Our findings also raise the question of whether other types of cargo are turned over in a FOXO3-dependent manner. In support of this possibility, we observed several genes involved in mitophagy, a selective form of autophagy in which mitochondria are recruited in to the autophagy pathway, as FOXO3 targets in this study (e.g. *Pink1*, *Bnip3* and *Bnip3l*). Mitophagy has been linked to Parkinson’s disease and many Parkinson’s associated genes function in mitophagy [38]. Of note, the FOXO3 target *Pink1* functions as a mitochondrial targeted serine/threonine kinase that directly phosphorylates the E3-ubiquitin ligase *Parkin* on the surface of mitochondria to target them to the autophagosome [39]. Work in *Drosophila* has shown that FOXO3 regulates this process in fly neurons [40], and other studies suggest that this function is conserved in mammals as well [41]. Together with our findings and evidence for FOXO-mediated α-synuclein turnover, these studies suggest that mitochondrial homeostasis may be an essential, direct, and conserved function of the FOXO family.

While we are still at the early stages of understanding how proteostasis supports healthy stem cell homeostasis, our findings show that this process is maintained by a genomic network under tight transcriptional control. Future studies will be needed to fully understand how to best modulate the network to optimize cellular quality control in disease states to promote aggregate clearance and reset tissue homeostasis.

## Methods

### Ethics statement

Mice were maintained and used for experimentation according to the protocol approved by the Brown University Institutional Animal Care and Use Committee.

### Mouse NSPC cultures

Primary mouse NSPCs were isolated as described previously [13, 16]. Briefly, adult NSPCs were isolated from 12 week old subventricular zone (SVZ) tissue and 4 animals were pooled per preparation. For dissociation, tissues were minced and incubated for 30 minutes at 37°C in 2.5U/ml Papain (Worthington), 1 U/ml DNase1 (Sigma), Dispase II (Stem Cell Technologies) in HBSS (Invitrogen). Tissue was manually dissociated by trituration and progenitors were purified using 25% and 65% Percoll gradients (Fisher Scientific). Neurospheres were cultured in growth media containing Neurobasal A (Invitrogen), 2% B27 (Invitrogen), penicillin/streptomycin/glutamine (Invitrogen), 20 ng/ml FGF2 and 20 ng/ml EGF (both from Peprotech). For passage, whole neurospheres were dissociated by brief 2–3 minute incubation in Accutase (Life Technologies) followed by manual trituration to obtain single cells. Triple knockout of FOXOs was performed in postnatal day 1–8 primary NSPCs isolated from whole forebrains.

### Lentivirus and adenovirus experiments

For lentivirus experiments, NSPCs were plated on poly-D-lysine at 50,000 cells/cm^2^ and infected with viral supernatants sixteen hours after plating. Cells were infected with a 1:1 ratio of growth media to viral supernatant. Each culture was infected with the transactivator FUW-rtTA together with either FUW-TetO-EV, FUW-TetO-FOXO3, or FUW-TetO-FOXO3-CA. Virus was removed after 24 hours and replaced with growth medium containing 2 μg/ml doxycycline (Sigma). Adenoviruses (Ad5-EV, Ad5-Cre, Ad5-GFP and Ad5-Cre-GFP) were purchased from the University of Iowa Viral Vector Core Facility. For infection, neurospheres were dissociated, plated as single cells at 50,000 cells/ml, and immediately infected. Cells were infected at a multiplicity of infection (MOI) of 100 unless otherwise indicated. Virus was removed after 24 hours.

### Network and motif analysis of ChIP-seq data

Network analysis was performed using String v10. The GO autophagy gene set was obtained from the AmiGO 2 Gene Ontology database [42]. Statistical analysis was performed using R. Motif analysis was performed using Homer findGenomeMotifs.pl [43].

### RT-qPCR

Total RNA was isolated using the QIAGEN RNeasy kit with on column DNase digestion (QIAGEN). 500 ng-1 μg RNA was used for reverse transcription, which was performed using the High Capacity cDNA Reverse Transcription Kit (Applied Biosystems). qPCRs were performed using Powerup SybrGreen Master Mix (Invitrogen) and run on an Applied Biosystems ViiA 7 Real-Time PCR System. See S2 Table for primer sequences used.

### Western blots

FOXO3 western blots were performed as described previously [44]. Cells were lysed in Triton lysis buffer (50mM Tris-HCL pH 7.5, 100mM NaCl, 0.5 mM EDTA, 0.4% Triton, 50 mM NaF, 40 mM b-glycerophosphate, 1mM sodium orthovanadate, 1mM PMSF, 0.055 units/ml aprotinin). For LC3 western blots, cells were lysed in 150 mM NaCl, 1.0% Triton, 0.5% Sodium Deoxycholate, 0.05% SDS, 50mM Tris-HCL pH 8 and a cOmplete Mini Protease Inhibitor Cocktail Tablet (Sigma) by shaking at 4°C for 30 minutes. Lysates were centrifuged at max speed for 7 minutes (15 minutes for LC3) and supernatants transferred to new tubes. Protein concentration was quantified on a Qbit using the Qbit Protein Assay Kit (Life Technologies), followed by the addition of Laemmli sample buffer (2% SDS, 10% glycerol, 5% β-meracaptoethanol, 63 mM Tris-HCl pH 6.8, bromophenol blue). All antibodies were diluted in 5% powdered milk or BSA and primary antibody incubations were performed overnight at 4°C. Primary antibodies and concentrations used: Rabbit anti-LC3B 1:1000 (Novus Biologicals NB600-1384), Rabbit anti-FOXO3 1:1000 (CST 75D8), Rabbit anti-FOXO1 1:1000 (Abcam ab12161), Rabbit anti-pS6 1:1000 (CST 4858S), Mouse anti-S6 1:1000 (CST 2317S), Rabbit anti-Pink1 1:1000 (Novus BC100-494) Mouse anti-Actin 1:5000 (Novus Biologicals (NB100-74340).

### Fluorescence activated cell sorting

FACS analysis was performed using a FACSCalibur Flow Cytometer (Becton Dickinson) and data were analyzed using Flowjo software (v10). The LC3 reporter was previously described [8]. For Bafilomycin experiments, cells were treated for two hours with 0.1 μM Bafilomycin A (Sigma).

### Proteostat staining

For aggregate detection, cells were stained with Proteosat (Enzo) according to the manufacturer’s specifications. For Rapamycin treatment, cells were treated with 200 μM Rapamycin for 24 hours. Cells were grown on poly-D-lysine coated coverslips and fixed with 4% PFA for 10 minutes followed by three washes with 1 x PBS. Cells were permeabilized with Permeabilization Solution, washed with PBS, and incubated in Proteostat Dual Detection Reagent for 30 minutes at room temperature. Cells were washed with PBS and imaged with a Zeiss Axiovert 200M Fluorescence Microscope, and images were processed with Image J to measure fluorescence intensity.

## Supporting information

S1 Table Complete lists of all autophagy genes (GO: 0006914) bound by FOXO3 in NSPCs. (XLSX)

S2 Table Primer sequences used in RT-qPCR experiments. (XLSX)

S3 Table Expression of FOXO3 autophagy targets in *Foxo3^-/-^* NSPCs relative to *Foxo3^+/+^* cells. (XLSX)

S4 Table Underlying numerical data for graphs included in the main and supplementary figures. (XLSX)

S1 Fig FOXO3 induces autophagy genes in NSPCs. RT-qPCR analysis of candidate target genes under basal conditions (infected with empty vector) or overexpressing constitutively active FOXO3. n = 3 experiments; Student’s t-test; *p < 0.05, p** < 0.01.
(TIF)

S2 Fig Ablation of FOXO transcription factors in Trifloxed NSPCs by Cre-recombinase. **(A)** Western blots showing FOXO3 and FOXO1 protein levels in Trifloxed NSPCs infected with empty vector (EV) or Cre-recombinase adenoviruses at various multiplicity of infections (MOIs). **(B)** RT-qPCR analysis of *Foxo* family members in Trifloxed NSPCs infected with adenoviruses carrying GFP or Cre-recombinase-GFP. **(C)** Expression of the direct FOXO3 autophagy targets in *Foxo3*^*-/-*^ NSPCs compared to *Foxo3*^*+/+*^ cells.
(TIF)

S3 Fig FOXO3 regulates mitophagy genes in NSPCs. **(A)** Overlap between FOXO3 ChIP-seq targets in NSPCs and mitophagy genes (GO:0000422; Fisher’s exact test). **(B)** Expression of selected mitophagy genes in wild type and FOXO-ablated (Trifloxed) NSPCs. **(C)** RT-qPCR analysis of a subset of mitophagy genes in NSPCs overexpressing FOXO3-CA. Fold change for (B) and (C) is relative to the EV control for the respective experiments. n = 3 experiments; Student’s t-test; *p < 0.05, **p < 0.01, ****p < 0.0001. **(D)** Western blot showing PINK1 protein levels in control (EV; empty vector) and FOXO-ablated NSPCs, and under basal, starvation (HBSS), and HBSS+BafA conditions. One representative experiment of three replicates is shown.
(TIF)

S4 Fig The mCherry-GFP-LC3 tandem reporter system. **(A)** Example images of the mCherry-GFP-LC3 tandem reporter under basal conditions, conditions that increase autophagic flux (2 hour HBSS treatment), and conditions that block autophagy (2 hour BafA treatment). **(B)** Quantification of the images in (A). Autophagosomes marked by GFP are mobilized by starvation, indicated by decreased GFP (HBSS, left panel), but overall autophagy is elevated under this condition (HBSS, center and right panels). BafA blocks autophagosome/lysosome fusion, indicated by strong induction of mCherry signal (center and right panels). n = 3 experiments; Student’s t-test; *p < 0.05, p** < 0.01.
(TIF)

S5 Fig FACS plots for the LC3 tandem reporter. **(A)** FACS plot showing LC3-GFP reporter expression in NSPCs basally, and shifted in response to starvation (2 hours HBSS). **(B-C)** LC3-GFP intensity under basal **(B)** and starvation **(C)** conditions in control (empty vector) and FOXO3-overexpressing cells. **(D)** LC3-GFP intensity in under starvation conditions in control cells (empty vector), or overexpressing either FOXO3 or CA-FOXO3. **(E-F)** LC3-mCherry expression in NSPCs is unchanged by FOXO3 overexpression under basal or starvation conditions. **(G-H)** FACS analysis of LC3-GFP in Trifloxed NSPCs infected with control adenovirus (empty vector; **(G)**) or Cre-recombinase (FOXO conditional KO; **(H)**) under basal conditions and treated with Bafilomycin A to block autophagic flux. **(I)** Starvation stress (HBSS) can induce autophagy independent of FOXO activity.
(TIF)
