# Supplementary figures and images for: FOXO3 directly regulates an autophagy network to functionally regulate proteostasis in adult neural stem cells
Source: PLoS Genet. 2019 Apr 11;15(4):e1008097. doi: 10.1371/journal.pgen.1008097 (PMC6478346; doi:10.1371/journal.pgen.1008097)

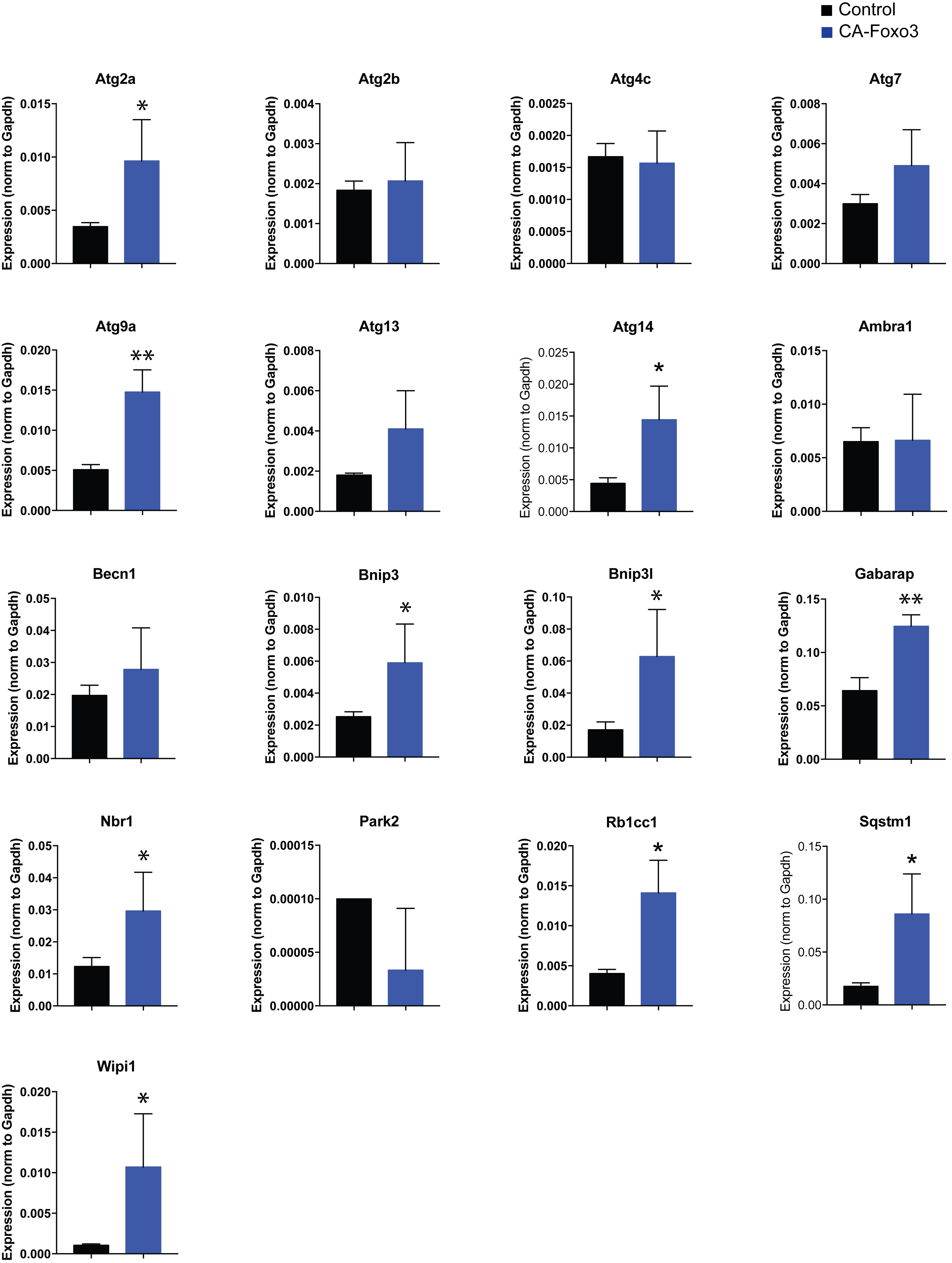

Supplement: S1 Fig — RT-qPCR analysis of candidate target genes under basal conditions (infected with empty vector) or overexpressing constitutively active FOXO3. n = 3 experiments; Student’s t-test; *p < 0.05, p** < 0.01. (TIF) [file pgen.1008097.s005.tif]

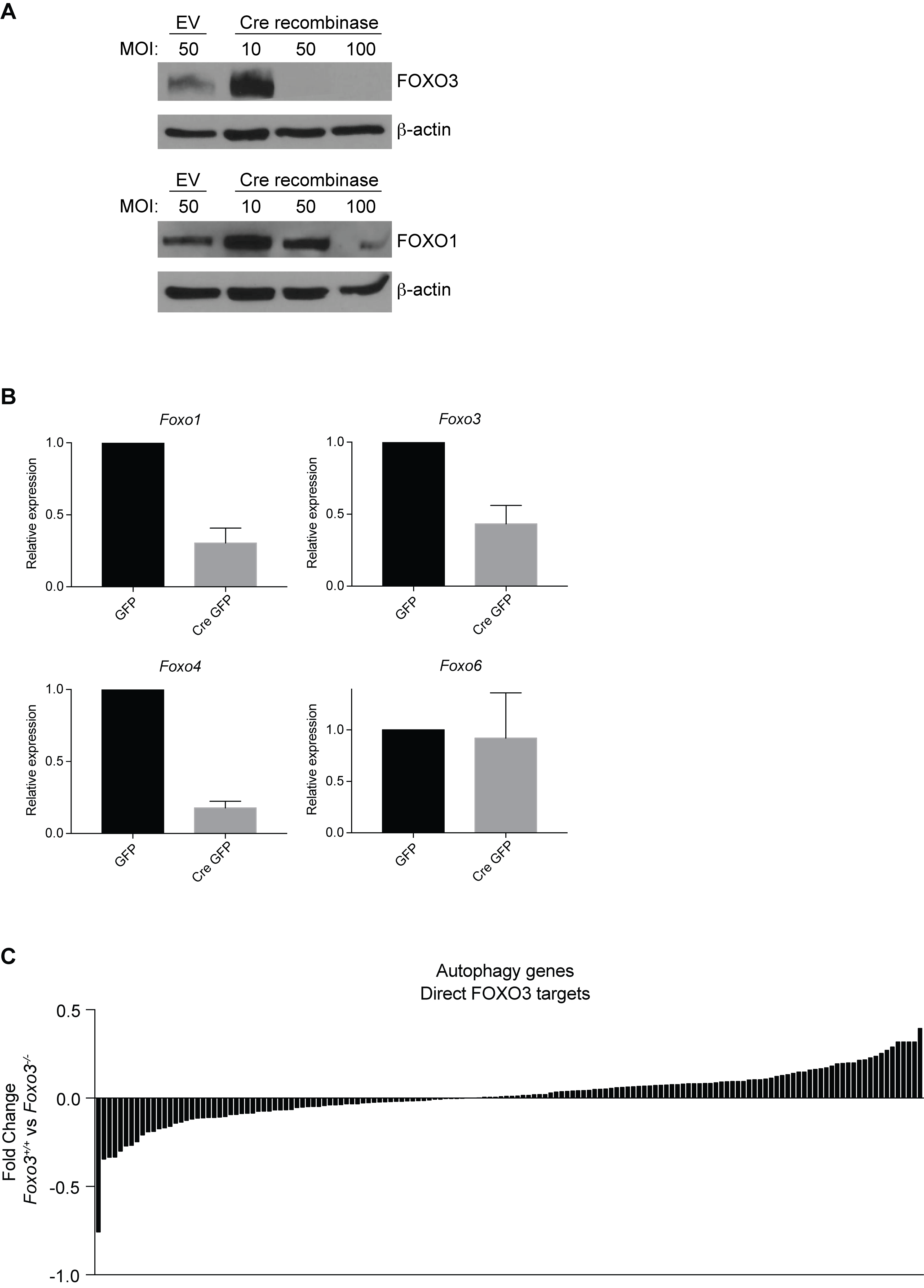

Supplement: S2 Fig — (A) Western blots showing FOXO3 and FOXO1 protein levels in Trifloxed NSPCs infected with empty vector (EV) or Cre-recombinase adenoviruses at various multiplicity of infections (MOIs). (B) RT-qPCR analysis of Foxo family members in Trifloxed NSPCs infected with adenoviruses carrying GFP or Cre-recombinase-GFP. (C) Expression of the direct FOXO3 autophagy targets in Foxo3-/- NSPCs compared to Foxo3+/+ cells. (TIF) [file pgen.1008097.s006.tif]

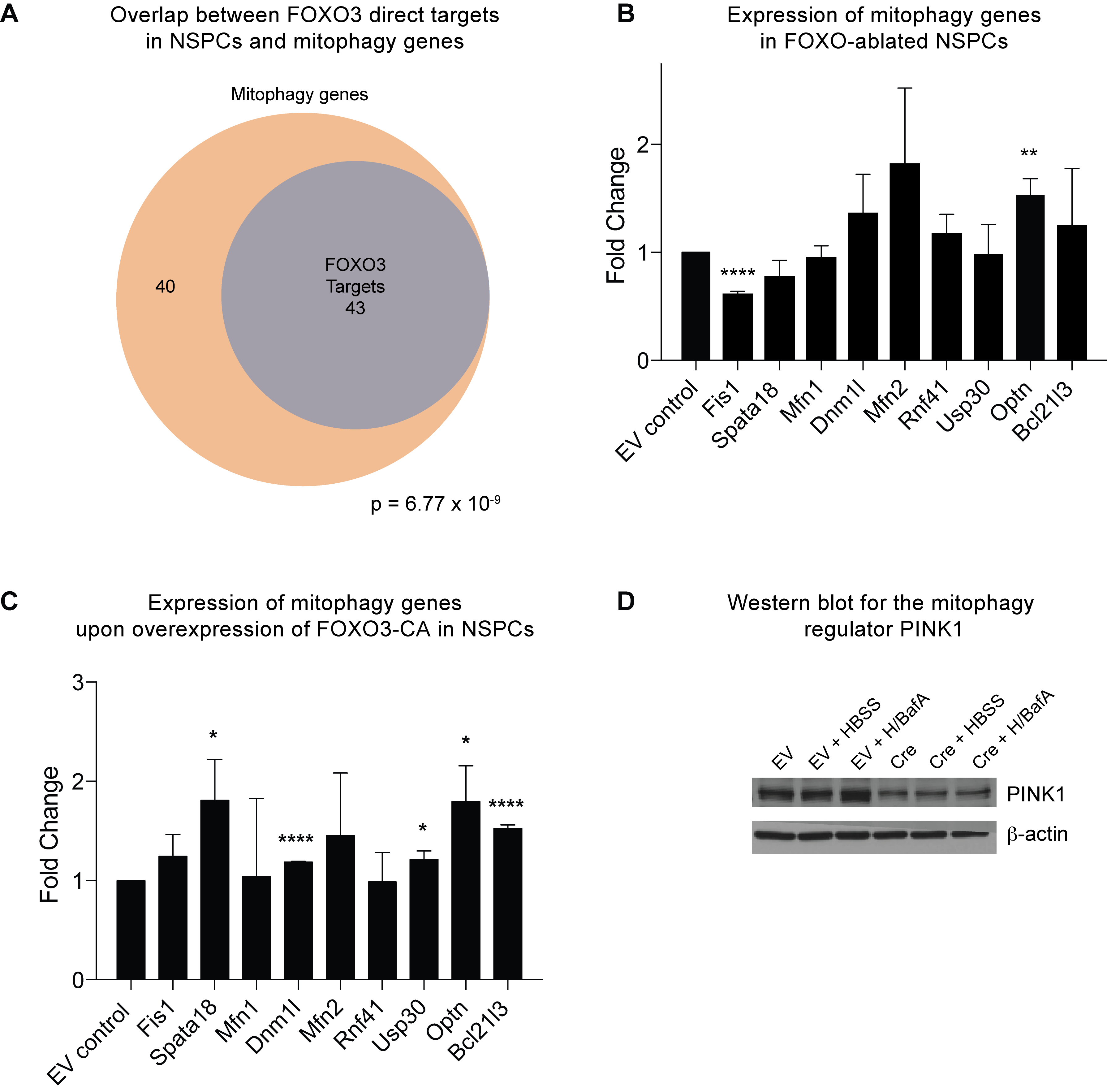

Supplement: S3 Fig — (A) Overlap between FOXO3 ChIP-seq targets in NSPCs and mitophagy genes (GO:0000422; Fisher’s exact test). (B) Expression of selected mitophagy genes in wild type and FOXO-ablated (Trifloxed) NSPCs. (C) RT-qPCR analysis of a subset of mitophagy genes in NSPCs overexpressing FOXO3-CA. Fold change for (B) and (C) is relative to the EV control for the respective experiments. n = 3 experiments; Student’s t-test; *p < 0.05, **p < 0.01, ****p < 0.0001. (D) Western blot showing PINK1 protein levels in control (EV; empty vector) and FOXO-ablated NSPCs, and under basal, starvation (HBSS), and HBSS+BafA conditions. One representative experiment of three replicates is shown. (TIF) [file pgen.1008097.s007.tif]

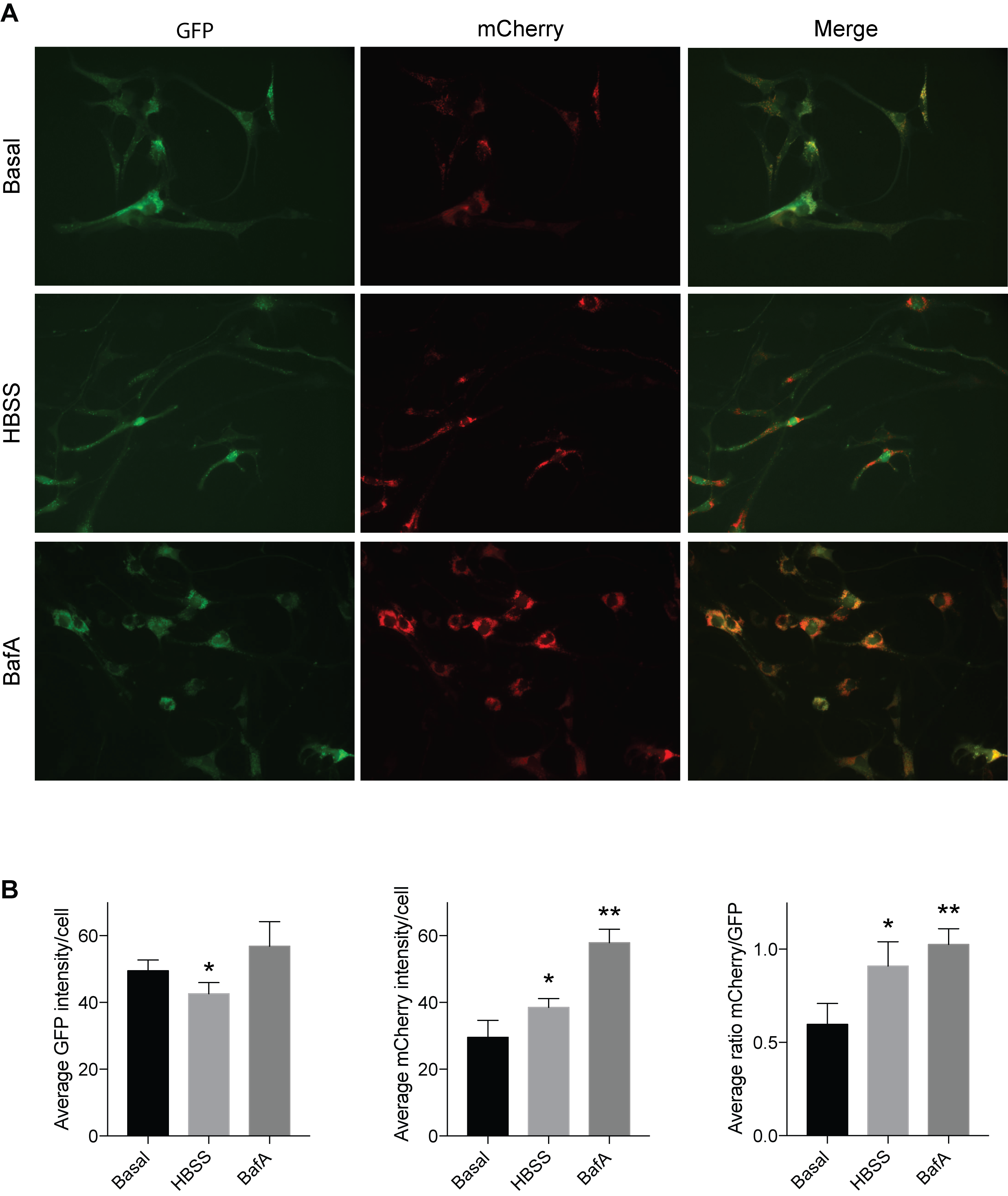

Supplement: S4 Fig — (A) Example images of the mCherry-GFP-LC3 tandem reporter under basal conditions, conditions that increase autophagic flux (2 hour HBSS treatment), and conditions that block autophagy (2 hour BafA treatment). (B) Quantification of the images in (A). Autophagosomes marked by GFP are mobilized by starvation, indicated by decreased GFP (HBSS, left panel), but overall autophagy is elevated under this condition (HBSS, center and right panels). BafA blocks autophagosome/lysosome fusion, indicated by strong induction of mCherry signal (center and right panels). n = 3 experiments; Student’s t-test; *p < 0.05, p** < 0.01. (TIF) [file pgen.1008097.s008.tif]

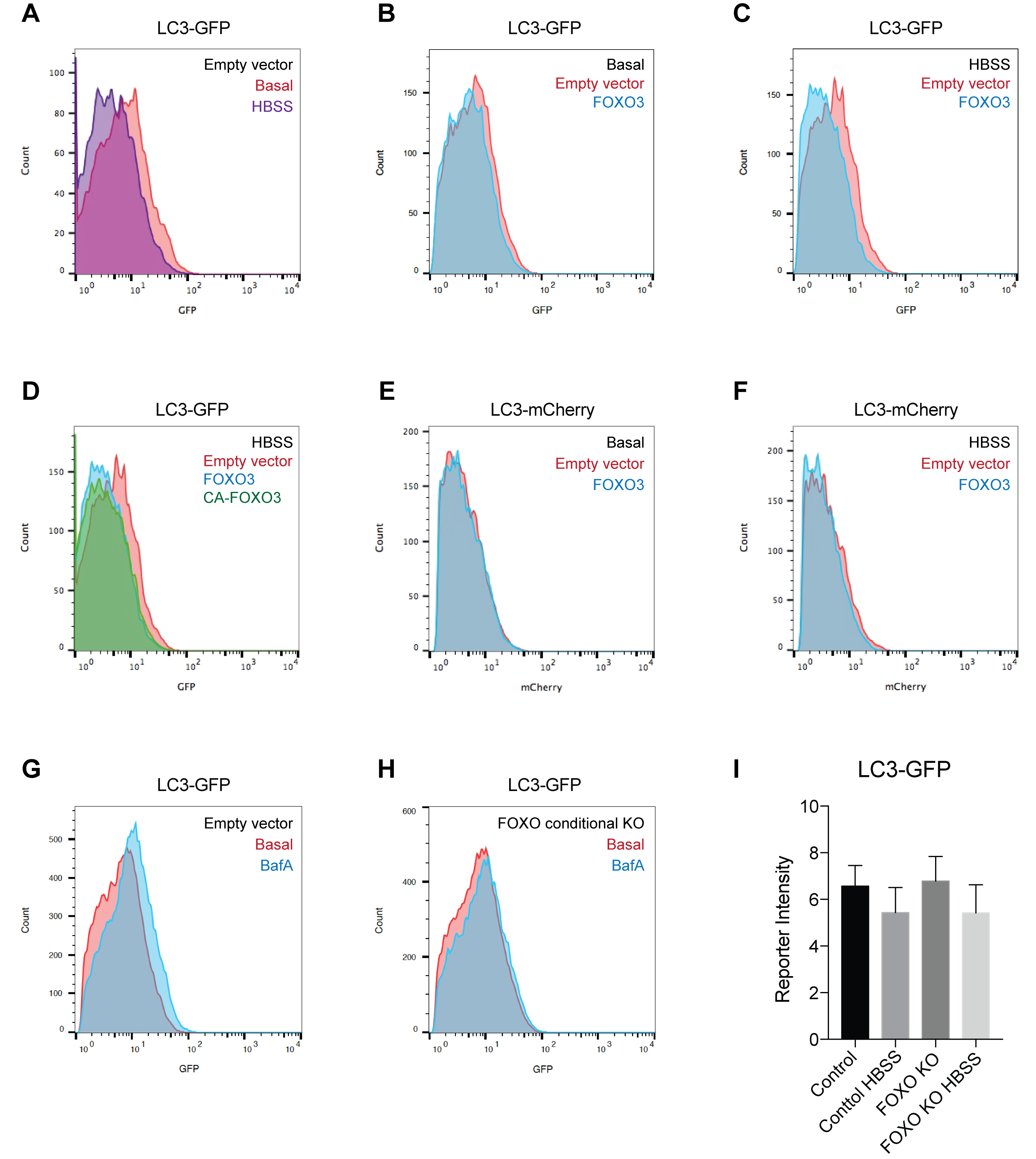

Supplement: S5 Fig — (A) FACS plot showing LC3-GFP reporter expression in NSPCs basally, and shifted in response to starvation (2 hours HBSS). (B-C) LC3-GFP intensity under basal (B) and starvation (C) conditions in control (empty vector) and FOXO3-overexpressing cells. (D) LC3-GFP intensity in under starvation conditions in control cells (empty vector), or overexpressing either FOXO3 or CA-FOXO3. (E-F) LC3-mCherry expression in NSPCs is unchanged by FOXO3 overexpression under basal or starvation conditions. (G-H) FACS analysis of LC3-GFP in Trifloxed NSPCs infected with control adenovirus (empty vector; (G)) or Cre-recombinase (FOXO conditional KO; (H)) under basal conditions and treated with Bafilomycin A to block autophagic flux. (I) Starvation stress (HBSS) can induce autophagy independent of FOXO activity. (TIF) [file pgen.1008097.s009.tif]
